# CKAP4 Antibody-Conjugated Si Quantum Dot Micelles for Targeted Imaging of Lung Cancer

**DOI:** 10.1186/s11671-021-03575-2

**Published:** 2021-07-31

**Authors:** Xin Huang, Qian Chen, Xin Li, Chenyu Lin, Kun Wang, Cici Luo, Wenjun Le, Xiaodong Pi, Zhongmin Liu, Bingdi Chen

**Affiliations:** 1 Shanghai East Hospital, The Institute for Biomedical Engineering and Nano Science, Tongji University School of Medicine, Shanghai, 200120 China; 2 State Key Laboratory of Silicon Materials and School of Materials Science and Engineering, Zhejiang University, Hangzhou, 310027 Zhejiang China

**Keywords:** Silicon, Quantum dot, CKAP4, Lung cancer, Fluorescence

## Abstract

At present, various fluorescent nanomaterials have been designed and synthesized as optical contrast agents for surgical navigation. However, there have been no reports on the preparation of fluorescent contrast agents for lung cancer surgery navigation using silicon quantum dots (Si QDs). This study improved and modified the water-dispersible Si QD micelles reported by Pi et al. to prepare Si QD micelles-CKAP4. The data showed that the Si QD micelles-CKAP4 were spherical particles with a mean hydrodiameter of approximately 78.8 nm. UV–visible absorption of the Si QD micelles-CKAP4 ranged from 200 to 500 nm. With an excitation wavelength of 330 nm, strong fluorescence at 640 nm was observed in the fluorescence emission spectra. Laser confocal microscopy and fluorescence microscopy assay showed that the Si QD micelles-CKAP4 exhibited good targeting ability to lung cancer cells and lung cancer tissues in vitro. The in vivo fluorescence-imaging assay showed that the Si QD micelles-CKAP4 was metabolized by the liver and excreted by the kidney. In addition, Si QD micelles-CKAP4 specifically targeted lung cancer tissue in vivo compared with healthy lung tissue. Cytotoxicity and hematoxylin and eosin staining assays showed that the Si QD micelles-CKAP4 exhibited high biosafety in vitro and in vivo. Si QD micelles-CKAP4 is a specifically targeted imaging agent for lung cancer and is expected to be a fluorescent contrast agent for lung cancer surgical navigation in the future.

**Supplementary Information:**

The online version contains supplementary material available at 10.1186/s11671-021-03575-2.

## Introduction

Cancer is one of the major diseases that threatens human health worldwide [1]. Among them, the incidence and mortality of lung cancer rank the highest among all malignant tumors and show an increasing trend every year [2]. Surgical resection is the primary treatment for solid tumors. However, the methods of differentiating malignant tissue from healthy tissue are mainly limited to tactile and visual cues and the surgeon’s experience [3].

In recent years, fluorescence-guided surgery has become popular in the research field in real-time intraoperative guidance for tumor resection [4]. With the help of a fluorescence contrast agent and fluorescence-imaging system, surgeons can use real-time image guidance to resect the primary tumors and identify residual tumors in the operating cavity. Compared with traditional biomedical imaging methods, fluorescence-imaging system offers several advantages, such as the absence of ionizing radiation and high spatial resolution [3, 5, 6].

At present, researchers have reported various fluorescent nanomaterials for biological imaging [7, 8]. Among them, silicon quantum dots (Si QDs) have become popular in the field of biological imaging [9–11]. However, there are no reports on the preparation of fluorescent contrast agents for lung cancer surgery navigation using Si QDs.

In 2014, Pi et al. reported a synthesis method for novel water-dispersible Si QD micelles [12]. The study data showed that Si QD micelles exhibited advantages including controllable size, high fluorescence quantum efficiency, and excellent light stability [12]. This study aims to improve and modify the water-soluble Si QD micelles reported by Pi et al. to prepare a novel fluorescent contrast agent, which is expected to be used as a fluorescent contrast agent for navigation in lung cancer surgery in the future.

In 2018, Yanagita et al. reported that CKAP4 is highly expressed in the serum and cancer tissues of lung cancer patients and it could be used as a new biomarker for the early diagnosis of lung cancer [13]. Therefore, CKAP4 antibody-conjugated Si QD micelles might be a novel fluorescent contrast agent, which might be used as a fluorescent contrast agent for navigation in lung cancer surgery.

In this study, DSPE-PEG-COOH was used to prepare Si QD micelles with carboxyl groups on their surfaces. We then conjugated the CKAP4 antibody with Si QD micelles using EDC. The results showed that the Si QD micelles-CKAP4 dispersed well in an aqueous solution. The Si QD micelles-CKAP4 exhibited strong red fluorescence under ultraviolet light irradiation at 365 nm. Due to the conjugation of antibody, Si QD micelles-CKAP4 showed good targeting ability to lung cancer cells and tissues both in vitro and in vivo. In addition, the Si QD micelles-CKAP4 exhibited high biosafety both in vitro and in vivo. In this study, we prepared a potential fluorescent contrast agent for navigation in lung cancer surgery.

## Methods

### Reagents

Bovine serum albumin (BSA) was purchased from Sigma (assay ≥ 98%; Missouri, USA); tetrahydrofuran (THF) from Adamas-beta (purity: 99.9%; Basel, Switzerland); DSPE-PEG-COOH from Aladdin (Shanghai, China); CKAP4 antibody from Abcam (0.55 mg/mL; Cambridge, UK); Dulbecco’s modified eagle’s medium (DMEM) and phosphate buffer saline (PBS) from Hyclone (Utah, USA); fetal bovine serum (FBS) and tyrisin from Gibco (New York, USA); paraformaldehyde (4%), immunostaining permeabilization buffer with Triton X-100 and blocking buffer for immunostaining from Beyotime (Shanghai, China); glycerin from Solarbio (purity ≥ 99%; Beijing, China); and trypsin from Kingmorn (0.25%; Shanghai, China).

### Instrumentation

Transmission electron microscopy (TEM) images were taken using JEM-2100F electron microscopy (JEOL, Tokyo, Japan). Hydrodiameter was determined using JEM Zetasizer Nano-ZS90 (Malvern Instruments, Malvern, UK). UV–Vis absorption spectra were obtained using Cary 50 spectrophotometer (Varian, Palo Alto, CA, USA). Fluorescence emission spectrum was obtained using F-182 4500 spectrophotometer (Hitachi Ltd., Tokyo, Japan). X-ray photoelectron spectroscopy (XPS) was performed using RBD-upgraded PHI-5000C ESCA system (Perkin Elmer, Waltham, MA, USA). Photoluminescence (PL) quantum yields (QYs) were estimated using fluorescent spectrometer (FLS1000, Edinburgh Instruments, Livingston, England). Fourier transform infrared spectroscopy (FTIR) was performed using Thermo IS50 (Thermo Fisher Scientific, Massachusetts, USA). X-ray diffraction (XRD) was tested by SmartLab (Rigaku Corporation, Tokyo, Japan).

### Cells

A549 and 293 T cell lines were purchased from the Shanghai Institute of Cell Biology (Shanghai, China).

### Preparation of Si QD Micelles

First, dodecly-Si QDs were prepared by a hydrosilylation reaction and dissolved in THF to prepare a dodecly-Si QDs solution (15 mg/mL) according to a previously reported method [12]. Then, 20 mg DSPE-PEG-COOH was dissolved and blended in 10 mL deionized water to prepare DSPE-PEG-COOH solution (2 mg/mL, 10 mL). Subsequently, a dodecly-Si QDs solution (15 mg/mL, 66 µL) was slowly added to a 10 mL DSPE-PEG-COOH solution dropwise. After ultrasonic agitation for 3 min, a clear and transparent solution was obtained. The solution was then placed in a fume hood and stirred for 12 h in the dark. Subsequently, the solution was dialyzed for 24 h and then freeze-dried for 3 days to obtain the Si QD micelles.

### Preparation of Si QD Micelles-CKAP4

In this study, CKAP4 antibody was conjugated with Si QD micelles by activating the carboxyl group by EDC [14]. The method was as follows: 4 mg of Si QD micelles was dispersed into 900 µL PBS to prepare the Si QD micelle solution. EDC (10 mg) was added to 100 µL of PBS to prepare the EDC solution. Then, 100 µL of EDC solution was slowly added to 900 µL of Si QD micelles, with ultrasonic agitation for 5 min. Furthermore, 20 µL of CKAP4 antibody was slowly added, and the solution mixture was stirred for 2 h at room temperature in the dark. The product was then ultrafiltered at 4000 rpm for 15 min. After washing with PBS three times, the product was referred to as Si QD micelles-CKAP4.

### Cytotoxicity Test

In this study, trypan blue was used to test the biosafety of the nanomaterials [15]. The A549 and 293 T cells were incubated in 24-well plate (1 × 10^6^/well) at 37 °C for 12 h. After discarding the medium, a series of concentrations of Si QD micelles or Si QD micelles-CKAP4 (Si concentration: 0–152 µg/mL) were added to a 24-well plate and incubated at 37 °C for 2 h. After discarding the medium, the cells were collected and washed twice with PBS. Trypan blue was blended with the cell suspension at a ratio of 1:1. The number of living and dead cells was detected and recorded using Countstar. Cell viability (%) = number of living cells/(number of living cells + number of dead cells) × 100. Three biological replicates were used. Prism software (version 7.01; GraphPad Software, San Diego, CA, USA) was used for statistical analysis. The data were analyzed using two-way ANOVA with Dunnett’s post test. Statistical significance was set at *P* < 0.05. *P < 0.05; **P < 0.01; and ***P < 0.001.

### Nanomaterials Target to Lung Cancer Cells In Vitro

The targeting ability of Si QD micelles and Si QD micelles-CKAP4 to lung cancer cells in vitro was detected as follows: First, A549 cells were incubated in laser confocal dishes (3 × 10^5^ cells/dish) at 37 °C for 12 h. After discarding the medium, the cells were washed three times with PBS. Subsequently, 1 mL paraformaldehyde (4%) was added to the cells and incubated at 25 °C for 10 min. After discarding the paraformaldehyde, the cells were washed with PBS three times. Next, 1 mL immunostaining permeabilization buffer with Triton X-100 was added to the cells and incubated at 25 °C for 10 min. After discarding the immunostaining permeabilization buffer with Triton X-100, cells were washed three times with PBS. Subsequently, 1 mL blocking buffer for immunostaining was added to the cells and incubated at 25 °C for 10 min. After discarding the blocking buffer for immunostaining, cells were washed three times with PBS. Then, 200 µL Si QD micelles or Si QD micelles-CKAP4 (Si concentration 150 μg/mL) were added to the cells and incubated at 37 °C for 2 h. After discarding the nanomaterials, the cells were washed three times with PBS. Photographs were taken using a laser confocal microscope (Leica, Wetzlar, Germany). The excitation light was set at 405 nm, and the emission wavelength was set at 630 nm.

The targeting ability of Si QD micelles and Si QD micelles-CKAP4 to normal cells in vitro was detected as follows: First, 293 T cells were collected in 1.5 mL Eppendorf tubes at a density of 1 × 10^6^ cells/tube. After centrifugation at 1000 rpm for 5 min, the supernatant was discarded. Next, 1 mL paraformaldehyde (4%) was added to the cells and incubated at room temperature for 10 min. After centrifugation at 1000 rpm for 5 min, the supernatant was discarded. The cells were then washed thrice with PBS. Then, 1 mL immunostaining permeabilization buffer with Triton X-100 was added to the cells and incubated at 25 °C for 10 min. The cells were then centrifuged at 1000 rpm for 5 min. After discarding the supernatant, the cells were washed three times with PBS. Then, 1 mL blocking buffer for immunostaining was added to the cells and incubated at 25 °C for 10 min. The cells were then centrifuged at 1000 rpm for 5 min. After discarding the supernatant, the cells were washed three times with PBS. Subsequently, 200 µL Si QD micelles or Si QD micelles-CKAP4 (Si concentration 150 μg/mL) were added to the cells and incubated at 37 °C for 2 h. After centrifugation at 1000 rpm for 5 min, the supernatant was discarded and the sediment was washed three times with PBS. The cells were harvested and microscope slides were prepared. Microscopy was performed using a laser confocal microscope. The excitation light was set at 405 nm, and the emission wavelength was set at 630 nm.

Three independent experiments were conducted. Quantitative analysis of mean fluorescence intensity was performed using ImageJ software (Bethesda, MD, USA). Prism software (version 7.01) was used for statistical analysis. The data were analyzed using one-way ANOVA with Dunnett’s post test. Statistical significance was set at *P* < 0.05. ***P < 0.001.

### Tumor-Bearing Mice Model

Ten male nude mice (5–6 weeks old) were purchased from Shanghai SLAC Laboratory Animal Co., Ltd. (Shanghai, China). All mice were housed under specific pathogen free (SPF) conditions at the Experimental Animal Center of Tongji University (Shanghai, China). To establish the tumor-bearing mouse model, mice were injected subcutaneously with 1 × 10^6^ A549 cells. Tumor-bearing mice were used in the experiment when the tumor diameter reached 6 mm. All mouse experiments were performed according to institutional guidelines and were approved by the Institutional Animal Use and Care Committee of Tongji University.

### Si QD Micelles-CKAP4 Targeting to Lung Cancer Tissues In Vitro

Tumor-bearing mice were sacrificed under anesthesia. The tumor and normal lung tissue were collected to prepare paraffin sections. After dehydration, antigen retrieval, and serum blocking, Si QD micelles or Si QD micelles-CKAP4 (Si concentration 150 µg/mL) were added to tissues and incubated at 37 °C for 2 h. After discarding the nanomaterials, the tissues were washed three times with PBS. The tissues were stained with 4′, 6-diamidino-2-phenylin-dole (DAPI) for 10 min. After washing three times with PBS, an antifade medium was added to the tissues and incubated at 25 °C for 5 min. After discarding the antifade medium, the tissues were washed three times with PBS. Photographs were taken using a fluorescence microscope (Nikon, Tokyo, Japan) with an excitation wavelength of 405 nm and an emission wavelength of 630 nm. Three independent experiments were conducted. A quantitative analysis of the mean red fluorescent intensity was performed using ImageJ software. Prism software (version 7.01) was used for statistical analysis. The data were analyzed using one-way ANOVA with Dunnett’s post test. Statistical significance was set at *P* < 0.05. ***P < 0.001.

### Fluorescence-Imaging In Vivo

In the experiment, nine tumor-bearing mice were divided into three groups (Si QD micelles-CKAP4 injection group, Si QD micelles injection group, and saline injection group). Mice in the Si QD micelles-CKAP4 injection group were intravenously injected with 200 µL of Si QD micelles-CKAP4 (Si: 4 mg/kg). Mice in the Si QD micelle injection group were intravenously injected with 200 µL of Si QD micelles (Si: 4 mg/kg). Mice in the saline injection group were intravenously injected with 200 µL saline. Fluorescence images were acquired at different time points (0, 0.25, 0.5, 1, 1.5, 2, 2.5, 3, 3.5, and 4 h). The mice in the Si QD micelles-CKAP4 injection group were anesthetized and euthanized at 4 h to detect the fluorescence signal in their heart, liver, spleen, lung, kidney, and tumor. In this study, fluorescence imaging was performed using a VISQUE Invivo Smart-LF (VIEWORKS, Gyeonggi-do, Korea) with a PE filter set (*λ*_excitation_ 450–500 nm, *λ*_emission_ 600–650 nm). Prism software (version 7.01) was used for statistical analysis. Data were analyzed using a one-way ANOVA with Dunnett’s post test. Statistical significance was set at *P* < 0.05. *P < 0.05; **P < 0.01.

### Hematoxylin and Eosin (H&E) Staining

In this study, 12 tumor-bearing mice were injected with saline (200 µL), Si QD micelles (Si: 4 mg/kg, 200 µL), or Si QD micelles-CKAP4 (Si: 4 mg/kg, 200 µL) through the tail vein. After 24 h, the mice were euthanized. H&E staining was performed on their hearts, livers, spleens, lungs, and kidneys. Pathological changes were observed by optical microscopy [14, 16].

## Results

### Preparation and Characterization of Si QD Micelles

**Scheme 1:**
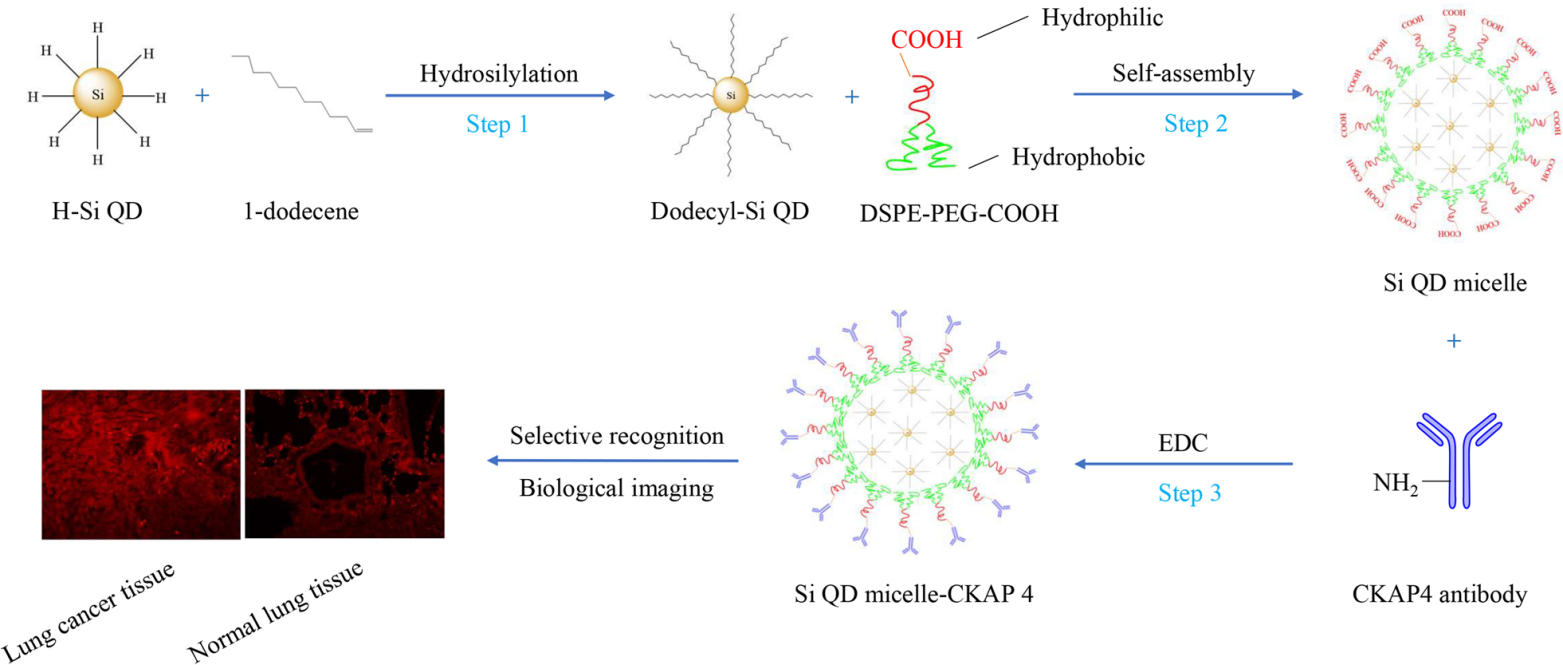
Schematic of Si QD micelles-CKAP4 preparation and targeted biological imaging in lung cancer tissues

**Fig. 1:**
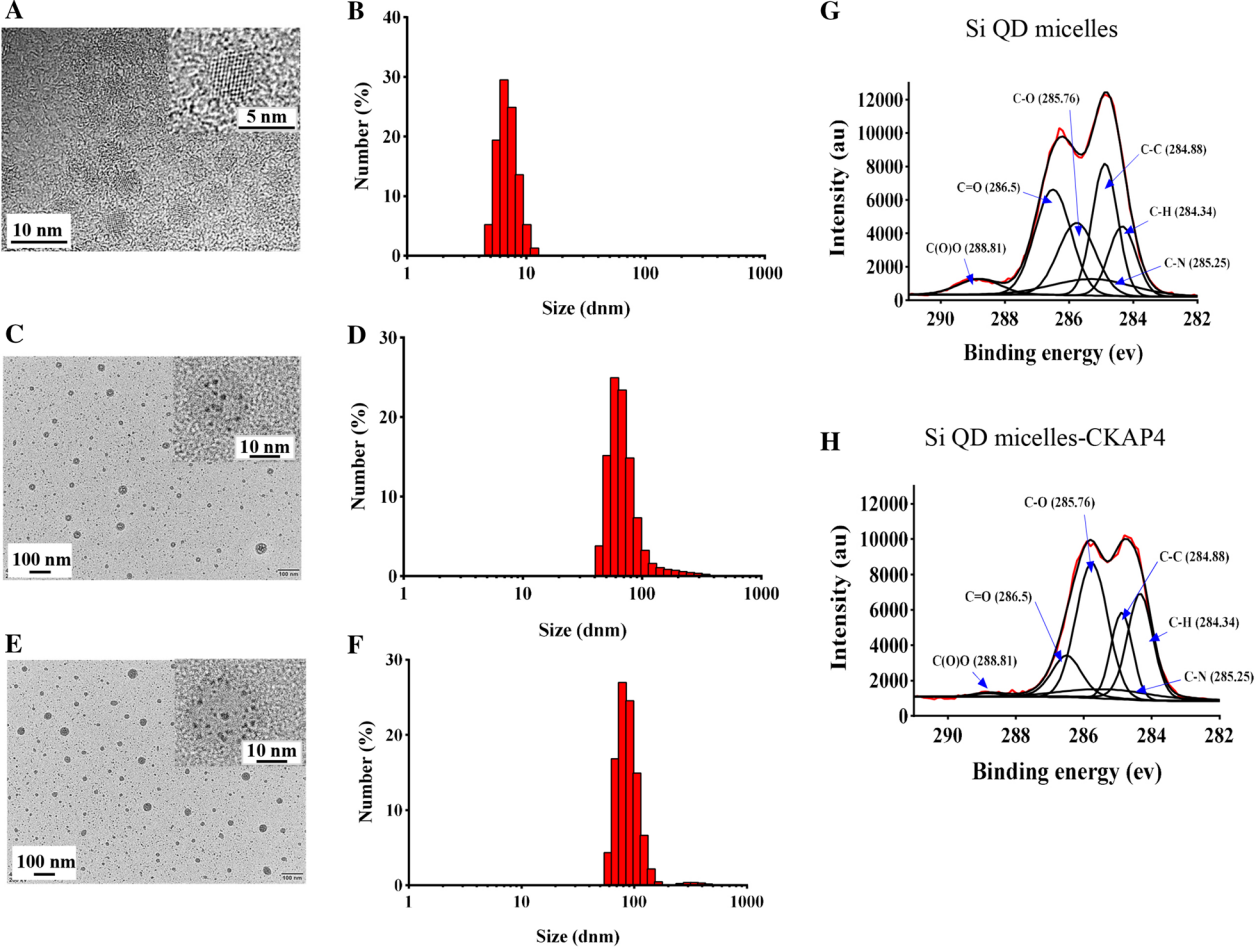
**a** TEM images of dodecyl-Si QDs; **b** hydrodiameter distribution of dodecyl-Si QDs; **c** TEM images of Si QD micelles; **d** hydrodiameter distribution of Si QD micelles; **e** TEM images of Si QD micelles-CKAP4; **f** hydrodiameter distribution of Si QD micelles-CKAP4; **g** C1s X-ray photoelectron spectroscopy (XPS) spectra of Si QD micelles; **h** C1s XPS spectra of Si QD micelles-CKAP4

First, dodecyl-Si QDs were prepared by hydrosilylation according to a previously published report (Scheme 1, Step 1) [12]. Similar to the data reported, dodecyl-Si QDs synthesized by our group dispersed well in methylbenzene [12]. TEM showed that the dodecyl-Si QDs were spherical with relatively uniform sizes. The sizes of the dodecyl-Si QDs in the TEM images were measured using ImageJ software. Data showed that the diameter of dodecyl-Si QDs was ranging from 4.1 to 5.3 nm, with an average diameter of about 4.7 nm (Fig. 1a). In the magnified images of the dodecyl-Si QDs, evident lattice fringes can be observed (Fig. 1a). In this study, the plane distance of the lattice was measured using the ImageJ software. Data showed that the average distance between lattice fringes is approximately 0.35 nm, which is nearly the same as that of the previously reported dodecyl-Si QDs (0.34 nm). Therefore, the dodecyl-Si QDs synthesized in this study showed good crystallinity [12]. Dynamic light scattering (DLS) showed the hydrodynamic diameters of dodecyl-Si QDs ranging from 4.8 to 13.5 nm with an average of 6.5 nm (Fig. 1b).

To prepare water-dispersible Si QD micelles, dodecyl-Si QDs were modified by self-assembly from DSPE-PEG-COOH (Scheme 1, Step 2) [12]. Here, DSPE-PEG-COOH was used instead of F127 to prepare Si QD micelles with carboxyl groups on the surface. The results showed that the Si QD micelles dispersed well in water. TEM images showed that the Si QD micelles were spherical particles with Si QDs inserted. The diameter of Si QD micelles was ranging from 22.7 to 54.6 nm, with an average diameter of approximately 34.3 nm (Fig. 1c). DLS showed that the hydrodynamic diameters of Si QD micelles ranged from 43.8 to 615.9 nm, with an average of 58.7 nm (Fig. 1d). The average zeta potential of the Si QD micelles was approximately − 20.7 mV. In this study, FTIR was performed to investigate the composition of the Si QD micelles (Additional file 1: Figure S1A). The data showed that the stretching vibration absorption peaks of N–H, C–H, O–H, and C=O were at 3430, 2920, 2850, and 1640 cm^−1^, respectively; the bending vibration absorption peaks of C–H and O–H were at 1460 and 1400 cm^−1^, respectively; the stretching vibration absorption peaks of Si–C, C–O, and P–O–C were at 1250, 1100, and 951 cm^−1^, respectively; the bending vibration absorption peaks of Si–H and O=C–N were at 850 and 526 cm^−1^, respectively. These data show that the characteristic groups of DSPE-PEG-COOH (such as N–H, O–H, C=O, C–O, P–O–C, and O=C–N) and the characteristic groups of dodecyl-Si QDs (such as Si–C and Si–H) could be found in the FTIR spectrum of Si QD micelles, indicating that dodecyl-Si QDs were successfully encapsulated in micelles self-assembled by DSPE-PEG-COOH. In addition, the Si QD micelles were characterized by XRD (Additional file 1: Figure S1B). The diffraction peak of the Si QD micelles was sharp, indicating that the crystallinity of the Si QD micelles was good. In the XRD pattern, peaks at 2*θ* of 27.073, 28.451, 56.598, and 73.145 correspond to the (100), (002), (112), and (203) crystal planes of silicon (PDF # 80-0005), indicating that the Si QDs were successfully encapsulated in the Si QD micelles. The peaks at 2*θ* of 31.692, 45.431, 66.200, 75.260, and 83.955 correspond to the (200), (220), (400), (420), and (422) crystal planes of NaCl (PDF # 77-2064). A rational explanation for the presence of NaCl in the Si QD micelles might be as follows: After Si QD micelle preparation, we dissolved Si QD micelles in PBS, leading to the presence of NaCl in the Si QD micelles.

**Fig. 2:**
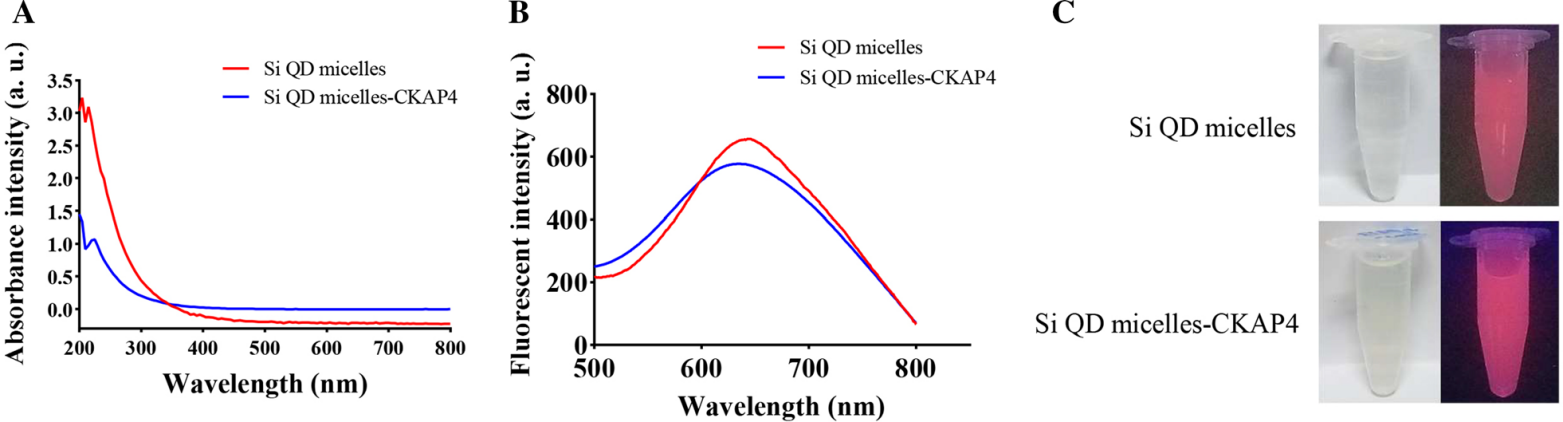
**a** UV–visible absorption spectrum of Si QD micelles and Si QD micelles-CKAP4; **b** fluorescent emission spectrum of Si QD micelles and Si QD micelles-CKAP4; **c** images of Si QD micelles and Si QD micelles-CKAP4 exposed in natural light (left) and 365 nm UV light (right)

UV–visible absorption spectrum showed that the absorption of Si QD micelles ranged from 200 to 500 nm (Fig. 2a). With an excitation wavelength of 330 nm, a strong fluorescence at 650 nm was observed in the fluorescence emission spectra of the Si QD micelles (Fig. 2b). The Si QD micelle aqueous solution (Si concentration: 80 µg/mL) was transparent and clear in natural light; however, it emitted strong red fluorescence under 365 nm UV light (Fig. 2c). In this study, the absolute PL QYs were tested using a fluorescent spectrometer (FLS1000). The excitation wavelength was 365 nm. The data showed that the absolute PL QY of the Si QD micelles was approximately 14.49%.

### Preparation and Characterization of Si QD Micelles-CKAP4

We conjugated the CKAP4 antibody with Si QD micelles using EDC (Scheme 1, Step 3) [14]. TEM images showed that the Si QD micelles-CKAP4 were spherical particles with Si QDs inserted. The diameter of Si QD micelles-CKAP4 ranged from 24.4 to 67.7 nm, with an average diameter of approximately 35.5 nm (Fig. 1e). DLS showed that the hydrodynamic diameters of the Si QD micelles-CKAP4 ranged from 58.7 to 824.9 nm with an average of 78.8 nm, which was slightly larger than that of Si QD micelles (Fig. 1f). This increase may be because of the conjugation of CKAP4 antibody. The C1s XPS spectra showed six types of carbon atoms in the C1s region: C(O)O (288.81 eV), C=O (286.5 eV), C–O (285.76 eV), C–N (285.25 eV), C–C (284.88 eV), and C–H (284.34 eV) (Fig. 1g). However, the peak of C(O)O nearly disappeared in the XPS spectra of the Si QD micelles-CKAP4, indicating that the conjugation between the carboxyl groups and primary amines was successful; therefore, the CKAP4 antibody was successfully conjugated with the Si QD micelles (Fig. 1h). The average zeta potential of the Si QD micelles-CKAP4 was approximately − 10.7 mV, higher than that of the Si QD micelles. The explanation for the above phenomenon might be as follows: There are many carboxyl groups on the surface of Si QD micelles; therefore, Si QD micelles exhibited strong negative electricity. However, in the Si QD micelles-CKAP4, the coupling of CKAP4 antibody led to the decrease of carboxyl groups on the surface of the Si QD micelles-CKAP4; therefore, the average zeta potential of the Si QD micelles-CKAP4 increased.

UV–visible absorption spectrum showed that the absorption of the Si QD micelles-CKAP4 ranged from 200 to 500 nm, similar to the result of Si QD micelles (Fig. 2a). With an excitation wavelength of 330 nm, a strong fluorescence at 640 nm was observed from Si QD micelles-CKAP4 in the fluorescence emission spectra (Fig. 2b). These results indicated that antibody conjugation exerted no significant influence on the fluorescence properties of the Si QD micelles-CKAP4. The Si QD micelles-CKAP4 aqueous solution (Si concentration: 80 µg/mL) was transparent and clear in natural light. Under 365 nm UV light, it could emit strong red fluorescence, consistent with the results of the Si QD micelles (Fig. 2c). PL QY tests showed that the absolute PL QY of the Si QD micelles-CKAP4 was approximately 16.96%.

### Cytotoxicity Test of Si QD Micelles-CKAP4 In Vitro

**Fig. 3:**
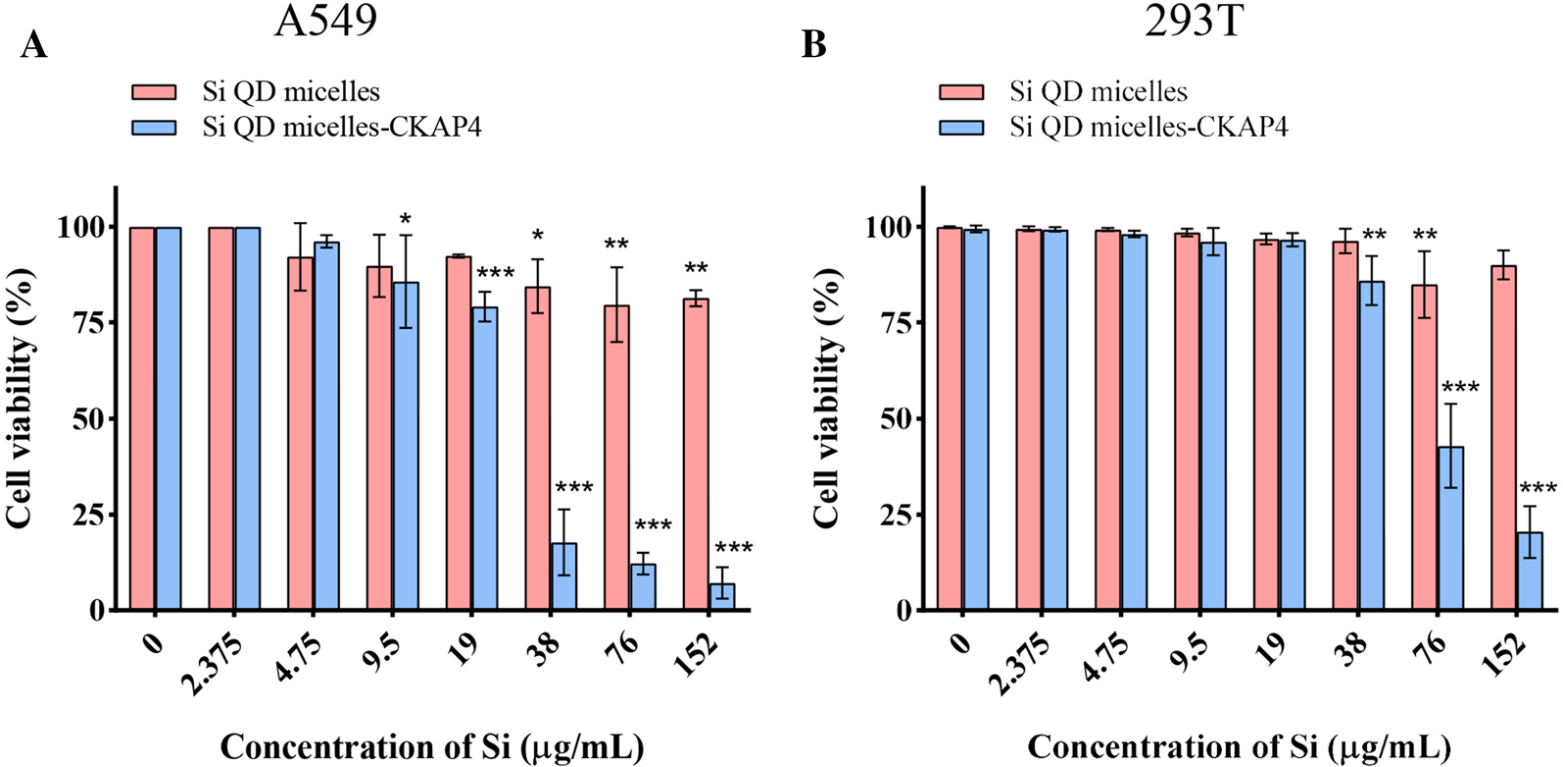
Cell viability of A549 **a** and 293 T **b** incubated with different concentrations of Si QD micelles and Si QD micelles-CKAP4 (Si concentration: 0, 2.375, 4.75, 9.5, 19, 38, 76, and 152 µg/mL) for 2 h (*n* = 3; mean ± SD; two-way ANOVA with Dunnett’s post test; *P < 0.05; **P < 0.01; and ***P < 0.001)

The human lung cancer cell line A549 and human renal epithelial cell line 293 T were used as target cells to investigate the biosafety of Si QD micelles and Si QD micelles-CKAP4. In this study, different concentrations of Si QD micelles and Si QD micelles-CKAP4 (Si concentration 0, 2.375, 4.75, 9.5, 19, 38, 76, and 152 µg/mL) were first incubated with A549 or 293 T cells at 37 °C for 2 h. Cell viability was detected using trypan blue (Fig. 3) [15]. The ID50 of Si QD micelles and Si QD micelles-CKAP4 was obtained using the GraphPad software. Data showed that the ID50 of Si QD micelles was greater than 152 µg/mL in A549 and 293 T cells. The ID50 of Si QD micelles-CKAP4 was 25.94 µg/mL in A549 cells and 72.69 µg/mL in 293 T cells. These results indicated that the cytotoxicity of Si QD micelles-CKAP4 was significantly higher than that of Si QD micelles and the cytotoxicity of Si QD micelles-CKAP4 in A549 cells was significantly higher than that of 293 T cells. This phenomenon may be because of complement-dependent cytotoxicity [17]. In addition, these results also indicated that Si QD micelles-CKAP4 showed higher biological safety in normal cells than in lung cancer cells, which laid a foundation for its application in vivo.

### Si QD Micelles-CKAP4 Targeting to Lung Cancer Cells In Vitro

**Fig. 4:**
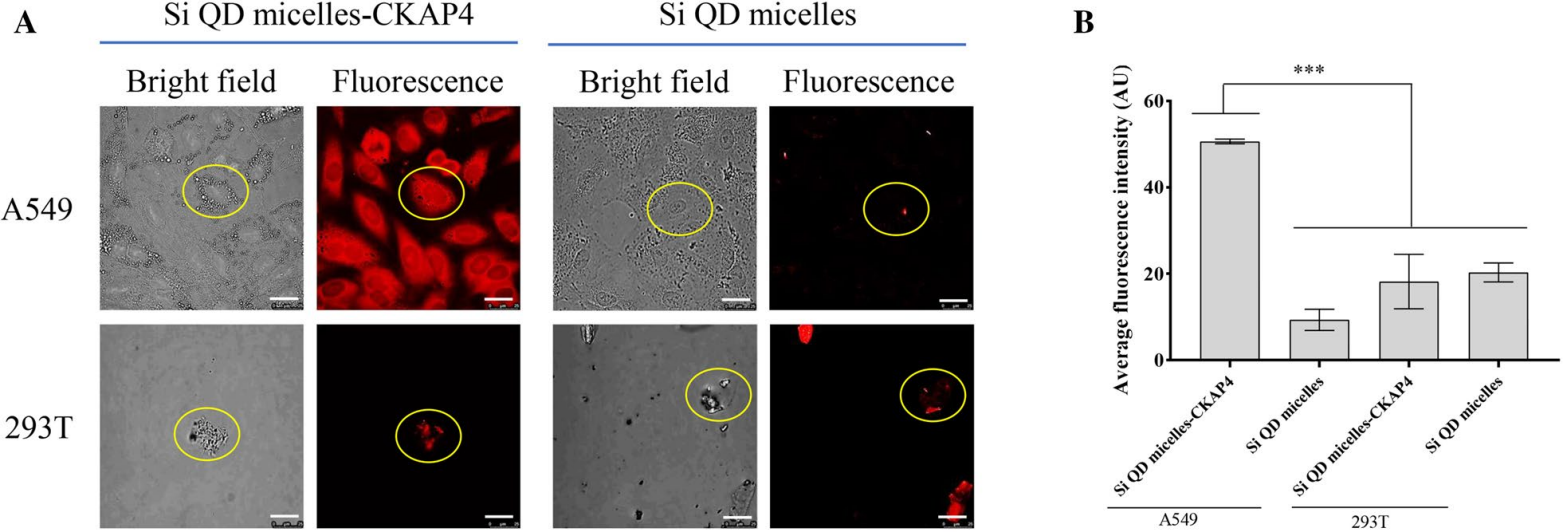
Detection of the capability of Si QD micelles-CKAP4 targeting to A549 cells in vitro. **a** A549 and 293 T were incubated with Si QD micelles-CKAP4 and Si QD micelles (the concentration of Si 150 µg/ml) at 37 ℃ for 2 h. After discarding the supernatant and washing twice, cells were pictured by confocal laser scanning microscope (Bar, 25 µm). **b** A quantitative analysis of mean fluorescent intensity was measured by ImageJ (*n* = 3; mean ± SD; one-way ANOVA with Dunnett’s post test; ***P < 0.001)

To further investigate the targeting function of Si QD micelles-CKAP4 in lung cancer cells in vitro, A549 and 293 T cells were used as target cells. Si QD micelles and Si QD micelles-CKAP4 were first incubated with A549 and 293 T cells at 37 °C for 2 h. The targeting effect was detected using laser confocal microscopy (Fig. 4). The results showed that the red fluorescence in the Si QD micelles-CKAP4 incubated A549 was significantly higher than that of the Si QD micelles-CKAP4 and 293 T co-incubating group, Si QD micelles and A549 co-incubating group, and Si QD micelles and 293 T co-incubating group. These results indicated that the Si QD micelles-CKAP4 could specifically target A549 cells in vitro.

### Si QD Micelles-CKAP4 Targeting to Lung Cancer Tissues In Vitro

**Fig. 5:**
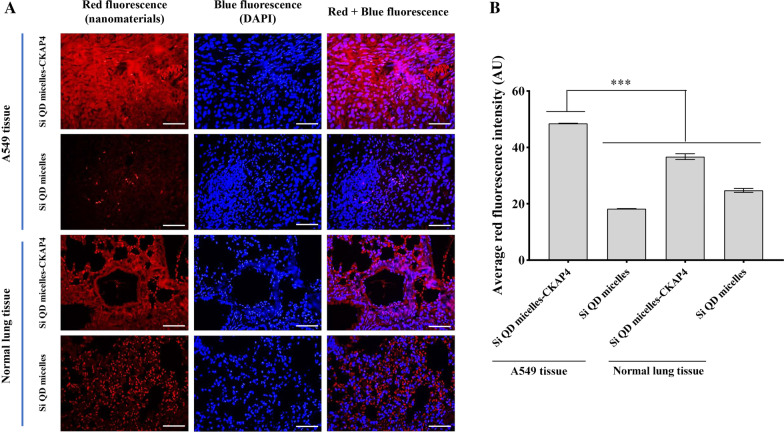
Detection of the capability of Si QD micelles-CKAP4 targeting to A549 tissue in vitro. **a** A549 tissue and normal lung tissue were cultured with Si QD micelles-CKAP4 and Si QD micelles (the concentration of Si: 150 µg/ml) at 37 ℃ for 2 h. After washing twice, tissues were stained with DAPI to visualize nucleus. Tissues were pictured by confocal laser scanning microscope (Bar, 200 µm). **b** A quantitative analysis of mean red fluorescent intensity was measured by ImageJ (*n* = 3; mean ± SD; one-way ANOVA with Dunnett’s post test; ***P < 0.001)

To explore the possibility of Si QD micelles-CKAP4 targeting lung cancer tissues, we designed an in vitro experiment to verify the targeting property of Si QD micelles-CKAP4 to A549 tissues. In this study, A549 cells were inoculated subcutaneously into the mice. After tumor formation, A549 tumor tissues and normal lung tissues were collected and incubated with Si QD micelles and Si QD micelles-CKAP4 at 37 °C for 2 h. The targeting effect was detected using laser confocal microscopy (Fig. 5). The results showed that Si QD micelles-CKAP4-incubated A549 tissue could emit strong red fluorescence at an excitation wavelength of 405 nm. However, only a weak red fluorescence was observed in Si QD micelle-incubated A549 tissues, Si QD micelles-CKAP4 incubated normal lung tissue, and Si QD micelles-incubated normal lung tissue. The red fluorescence in the Si QD micelles-CKAP4 incubated A549 tissue was significantly higher than that of Si QD micelles-incubated A549 tissues, Si QD micelles-CKAP4 incubated normal lung tissue, and Si QD micelles-incubated normal lung tissue. These data indicate that the Si QD micelles-CKAP4 could effectively target lung cancer tissues in vitro, which is supposed to be a potential fluorescent contrast agent for lung cancer.

### Fluorescence-Imaging In Vivo

Nine tumor-bearing mice were divided into three groups (Si QD micelles-CKAP4 injection group, Si QD micelles injection group, and saline injection group). Mice in the Si QD micelles-CKAP4 injection group were intravenously injected with 200 µL Si QD micelles-CKAP4 (Si: 4 mg/kg). Mice in the Si QD micelle injection group were intravenously injected with 200 µL of Si QD micelles (Si: 4 mg/kg). Mice in the saline injection group were intravenously injected with 200 µL saline. Fluorescence images were acquired at different time points (0, 0.25, 0.5, 1, 1.5, 2, 2.5, 3, 3.5, and 4 h). The significant fluorescence signals were not observed in lung cancer tissues among all the mice in the three groups (data not shown). This might be because of the weak fluorescence penetration of the Si QD micelles-CKAP4 and Si QD micelles.

**Fig. 6:**
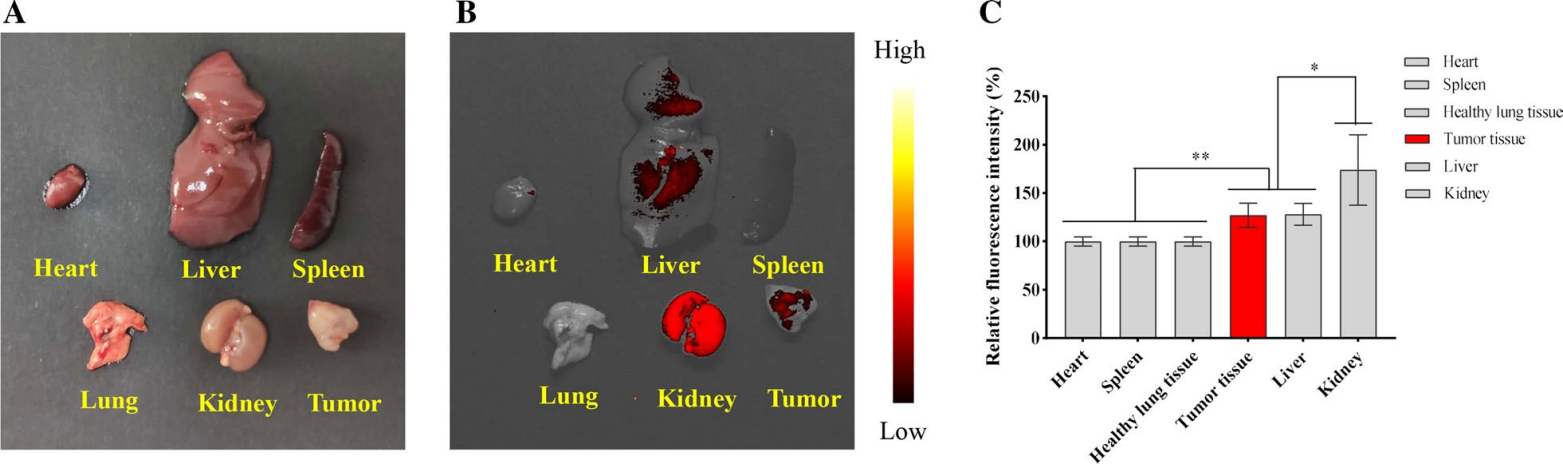
**a** Bright photograph of the heart, liver, spleen, lung, kidney, and tumor in Si QD micelles-CKAP4 injected mice. **b** Fluorescence photograph of the heart, liver, spleen, lung, kidney, and tumor in Si QD micelles-CKAP4 injected mice. **c** Relative fluorescence intensities of heart, liver, spleen, lung, kidney and tumor tissue (*n* = 3; mean ± SD; one-way ANOVA with Dunnett’s post test; *P < 0.05; **P < 0.01)

The mice in the Si QD micelles-CKAP4 injection group were anesthetized and euthanized at 4 h to detect the fluorescence signal in their heart, liver, spleen, lung, kidney, and tumor. Data showed that a significant fluorescence signal could be observed in the liver, kidney, and tumor tissues. However, there was no significant fluorescence signal in the heart, spleen, or healthy lung tissues (Fig. 6). The significant fluorescence signal in the liver and kidney indicated that the Si QD micelles-CKAP4 was metabolized by the liver and excreted by the kidney. In addition, the fluorescence signal in A549 tumor tissues was significantly higher than that in healthy lung tissues. This phenomenon contributed to the targeting ability of the Si QD micelles-CKAP4 to lung cancer tissues. These results indicate that Si QD micelles-CKAP4 specifically targets lung cancer tissue, which is expected to be a fluorescent contrast agent for lung cancer surgical navigation in the future.

### Biosafety Evaluation of Si QD Micelles-CKAP4 In Vivo

**Fig. 7:**
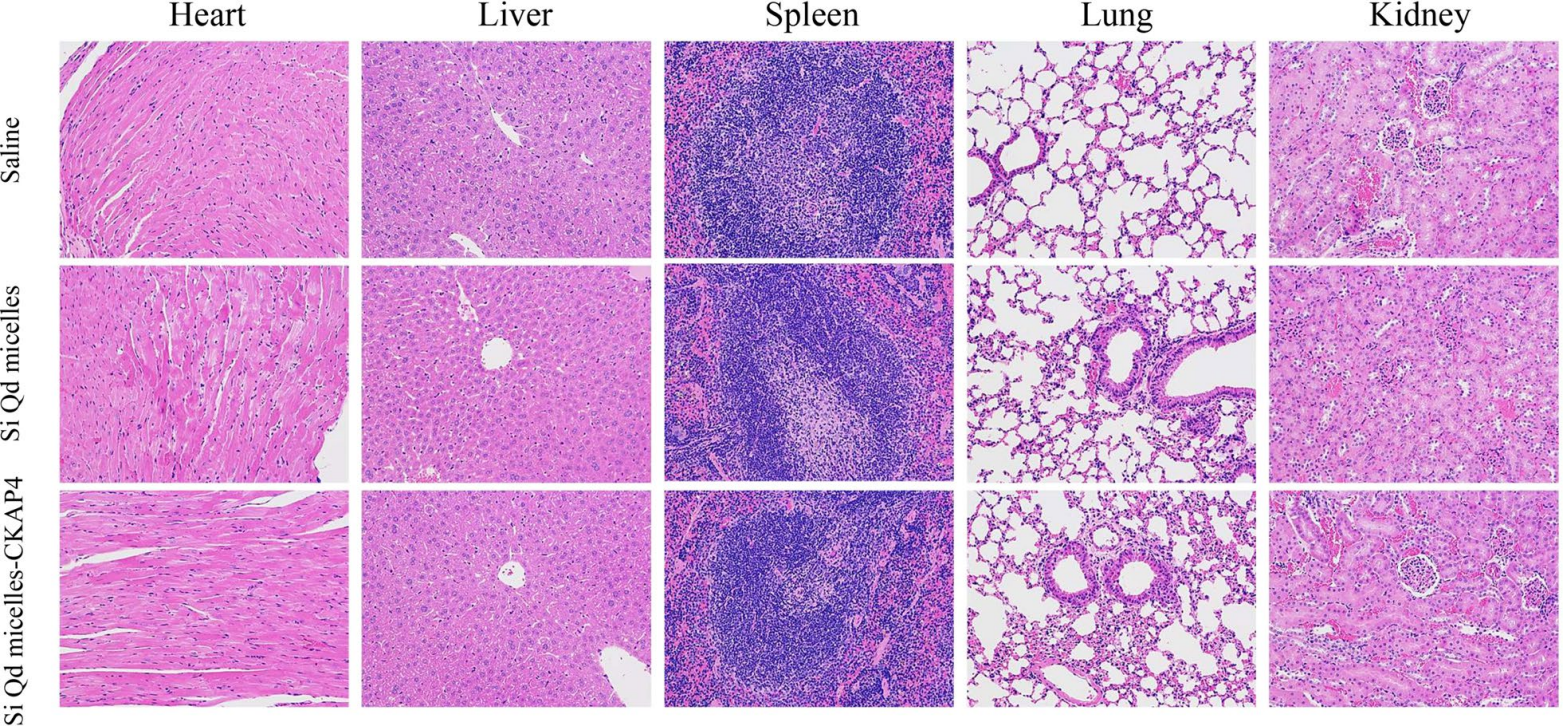
H&E staining images of the heart, liver, spleen, lungs, and kidneys in tumor-bearing mice injected with saline, Si QD micelles (Si: 4 mg/kg), and Si QD micelles-CKAP4 (Si: 4 mg/kg) for 24 h (magnification: 200×)

Finally, we investigated the acute toxicity of the Si QD micelles-CKAP4 in vivo. In this study, tumor-bearing mice (*n* = 12) were intravenously injected with saline (200 µL), Si QD micelles (Si: 4 mg/kg, 200 µL), or Si QD micelles-CKAP4 (Si: 4 mg/kg, 200 µL) and then euthanized 24 h later. H&E staining images of the heart, liver, spleen, lungs, and kidneys are shown in Fig. 7. Compared with saline-injected mice, there were no evident pathological changes in the major organs of Si QD micelles-injected mice and Si QD micelles-CKAP4-injected mice. The above data indicated that Si QD micelles (Si: 4 mg/kg) and Si QD micelles-CKAP4 (Si: 4 mg/kg) showed no significant toxicity in vivo.

## Discussion

At present, various fluorescent nanomaterials have been designed and synthesized as optical contrast agents for surgical navigation [3, 4]. In 2020, On et al. prepared fluorophore [indocyanine green (ICG) or cyanine 5.5 (Cy5.5)]-conjugated glycol chitosan nanoparticles (CNPs) as a fluorescent contrast agent in lung cancer surgery navigation [18]. However, there are no reports on the preparation of fluorescent contrast agents for lung cancer surgery navigation using Si QDs.

This study successfully synthesized Si QD micelles-CKAP4, which could target the imaging of lung cancer tissues. Si QD micelles-CKAP4 is expected to be used as an optical contrast agent for navigation in lung cancer surgery in the future. The advantages of Si QD micelles-CKAP4 are as follows: (1) We synthesized a fluorescent contrast agent for lung cancer surgery navigation using Si QDs for the first time, which expands the toolbox of the lung cancer surgery navigation tool library. (2) This study optimized water-dispersible Si QD micelles reported by Pi et al. to add targeting ability to Si QD micelles [12]. Thus, it provides a new possibility for applying Si QDs in the biomedical field. (3) The principle of Si QD micelles-CKAP4 targeting lung cancer is based on the high expression of CKAP4 protein in lung cancer tissue. Owing to the conjugation of CKAP4 antibody, Si QD micelles-CKAP4 could actively target lung cancer tissue. Compared with the nanomaterials that passively target tumors via the EPR effect, Si QD micelles-CKAP4 showed stronger specificity to lung cancer tissue. (4) Si QD micelles-CKAP4 showed selective toxicity to tumor cells in a specific range of concentrations. Therefore, Si QD micelles-CKAP4 showed high biological safety in the range of rational drug concentrations, which exhibited its potential clinical application value.

In this study, PL QY tests showed that the absolute PL QY of Si QD micelles was approximately 14.49% and the absolute PL QY of the Si QD micelles-CKAP4 was approximately 16.96%. A study by Pi et al. showed that the PL QY of dodecyl-Si QDs was approximately 26%, which was very high for Si QDs that emit red light with a PL peak at less than 700 nm [12, 19, 20]. However, the PL QY of the Si QD micelles in this study was approximately 14.49%, which was lower than that of dodecyl-Si QDs. This might be because of the surface defects introduced during the emulsion process, which was nearly consistent with the decrease in PL QY in Si-QD micelles (dodecyl-passivated Si QDs were encapsulated in the micelles self-assembled from F127 in the emulsion) in the study reported by Pi et al. [12, 21]. In this study, the PL QY of Si QD micelles-CKAP4 was approximately 16.96%, which was slightly higher than that of Si QD micelles (14.49%). This could be because in the Si QD micelles-CKAP4, the coupling of the CKAP4 antibody repaired some surface defects of the Si QD micelles, leading to a slight enhancement of the PL QY.

As we all know, there is a challenge in Si QDs biological imaging applications: Although Si exhibits little cytotoxicity, Si particles may exhibit cytotoxicity as the size decrease to a certain extent. In this study, cytotoxicity tests showed that Si QD micelles-CKAP4 exhibited selective cytotoxicity to lung cancer cells at 9.5–19 µg/mL. Therefore, as a result of CKAP4 antibody conjugation, the cytotoxicity of Si QD micelles-CKAP4 to normal cells was significantly less than that of lung cancer cells, which provides a powerful condition for the biological application of Si QD micelles-CKAP4.

In this study, human lung cancer cell line A549 and human renal epithelial cell line 293 T were used as target cells to investigate the targeting ability of Si QD micelles and Si QD micelles-CKAP4. A549 and 293 T cells were adherent and semi-adherent, respectively. During the experiment, the number of 293 T cells decreased because of repeated centrifugation and washing. Therefore, as shown in Fig. 4, the number of 293 T cells was lower than that of A549 cells.

In addition, it should be noted that A549 cells belong to the p53 wild-type lung cancer cell line. Therefore, only A549 cells cannot fully reflect the targeting ability of the Si QD micelles-CKAP4 to lung cancer. Further studies will be conducted to test the targeting ability and imaging effect of Si QD micelles-CKAP4 on p53-negative cell lines (such as H1299) to provide a solid experimental basis for the clinical application of Si QD micelles-CKAP4 [22].

## Conclusions

This study improved and modified the Si QD micelles reported by Pi et al. to prepare water-dispersible Si QD micelles-CKAP4. Si QD micelles-CKAP4 were spherical particles with a mean hydrodiameter of approximately 78.8 nm. UV–visible absorption of the Si QD micelles-CKAP4 ranged from 200 to 500 nm. With an excitation wavelength of 330 nm, strong fluorescence at 640 nm was observed in the fluorescence emission spectra. Si QD micelles-CKAP4 exhibited good targeting ability to lung cancer cells and lung cancer tissues both in vitro and in vivo. In addition, the in vivo fluorescence-imaging assay further showed that the Si QD micelles-CKAP4 was metabolized by the liver and excreted by the kidney. Cytotoxicity and H&E staining assays showed that the Si QD micelles-CKAP4 exhibited high biosafety both in vitro and in vivo. Si QD micelles-CKAP4 is a specifically targeted imaging agent for lung cancer and is expected to be a fluorescent contrast agent for lung cancer surgical navigation in the future.

## Supplementary Information

**Additional file 1**. FTIR and XRD spectrum of Si QD micelles.

## Data Availability

The data and the analysis in the current work are available from the corresponding authors on reasonable request.

## Abbreviations

Si QD: Si quantum dot
Si QD micelles-CKAP4: CKAP4 antibody-conjugated Si quantum dot micelles
Dodecyl-Si QDs: Dodecyl-passivated Si QDs
BSA: Bovine serum albumin
THF: Tetrahydrofuran
DMEM: Dulbecco’s modified eagle’s medium
PBS: Phosphate buffer saline
FBS: Fetal bovine serum
TEM: Transmission electron microscopy
DLS: Dynamic light scattering
XPS: X-ray photoelectron spectroscopy
FTIR: Fourier transform infrared spectroscopy
XRD: X-ray diffraction
SPF: Specific pathogen free
DAPI: 4′, 6-Diamidino-2-phenylin-dole
H&E: Hematoxylin and eosin
