# Supplementary material for: CKAP4 Antibody-Conjugated Si Quantum Dot Micelles for Targeted Imaging of Lung Cancer
Source: Nanoscale Res Lett. 2021 Jul 31;16:124. doi: 10.1186/s11671-021-03575-2 (PMC8325747; doi:10.1186/s11671-021-03575-2)
Supplement: Supplementary file 1 — Additional file 1. FTIR and XRD spectrum of Si QD micelles. [file 11671_2021_3575_MOESM1_ESM.docx]

**CKAP4 Antibody Conjugated Si Quantum Dot Micelles for Targeted Imaging of Lung Cancer**

Xin Huang^1,†^, Qian Chen^1,†^, Xin Li^1^, Chenyu Lin^1^, Kun Wang^2^, Cici Luo^1^, Wenjun Le^1^, Xiaodong Pi^2^, Zhongmin Liu^1,*^, Bingdi Chen^1,*^


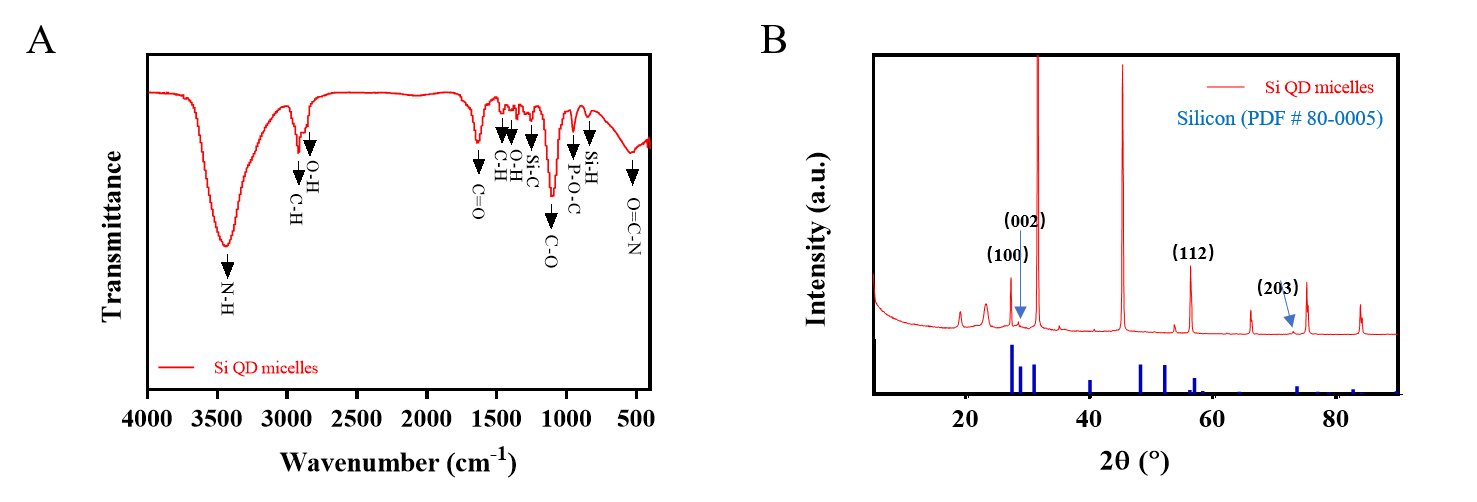


**Figure S1.** (A) FTIR spectrum of Si QD micelles; (B) XRD pattern of Si QD micelles.
